# The Influence of Electric Field Intensity and Particle Length on the Electrokinetic Transport of Cylindrical Particles Passing through Nanopore

**DOI:** 10.3390/mi11080722

**Published:** 2020-07-25

**Authors:** Liuyong Shi, Xiaohan He, Jian Ge, Teng Zhou, Ting Li, Sang Woo Joo

**Affiliations:** 1 Mechanical and Electrical Engineering College, Hainan University, Haikou 570228, China; shiliuyong@hainanu.edu.cn (L.S.); poisonzotn_h@163.com (X.H.); gejian@hainanu.edu.cn (J.G.); 2 Institute of Biomedical Engineering, Chinese Academy of Medical Sciences and Peking Union Medical College, Tianjin 300192, China; liting@bme.cams.cn; 3 School of Mechanical Engineering, Yeungnam University, Gyongsan 38541, Korea

**Keywords:** nanopore, nanofluidics, electrokinetics, mechanism, arbitrary Lagrangian–Eulerian (ALE)

## Abstract

The electric transport of nanoparticles passing through nanopores leads to a change in the ion current, which is essential for the detection technology of DNA sequencing and protein determination. In order to further illustrate the electrokinetic transport mechanism of particles passing through nanopores, a fully coupled continuum model is constructed by using the arbitrary Lagrangian–Eulerian (ALE) method. The model consists of the electric field described by the Poisson equation, the concentration field described by Nernst–Planck equation, and the flow field described by the Navier–Stokes equation. Based on this model, the influence of imposed electric field and particle length on the electrokinetic transport of cylindrical particles is investigated. It is found firstly the translation velocities for the longer particles remain constant when they locate inside the nanopore. Both the ion current blockade effect and the ion current enhancement effect occur when cylindrical particles enter and exit the nanopore, respectively, for the experimental parameters employed in this research. Moreover, the particle translation velocity and current fluctuation amplitude are dominated by the electric field intensity, which can be used to adjust the particle transmission efficiency and the ion current detectability. In addition, the increase in particle length changes the particle position corresponding to the peak value of the ion current, which contributes to distinguishing particles with different lengths as well.

## 1. Introduction

With the completion of the Human Genome Project, the era of post genomics is coming [1]. An urgent demand has been proposed for cheaper and faster DNA sequencing, and thus a new generation of DNA sequencing technology based on the nanopore has been developed rapidly [2,3,4]. Currently, there are two kinds of DNA sequencing technology based on nanopores: optical detection and electrical detection. Optical detection is to hybridize nucleotide and marker, and the marker can be detected when DNA is translocated so as to achieve sequencing [5,6]. Electrical detection mainly includes three detection methods, namely ion current [7,8], tunnel current [9,10], and potential difference [11,12]. Their basic principle is that when DNA molecules pass through the nanopore, they will temporarily affect the conductivity of the nanopore. The sequence of the base pairs can be distinguished based on the different influence of the base pairs on the ion current, the tunnel current and the potential difference between the two ends of the nanopore.

Generally, two kinds of nanopores are employed in the aforementioned analysis and detection technology. One is a biological nanopore, and the other is a solid nanopore. In a pioneering study of biological nanopores [13], α-hemolysin was employed to explore the movement of single-stranded DNA in 1996. It was found that the current blockade time was proportional to the length of the polymerization chain when single-stranded DNA molecules passed through the double-layer membrane structure with a diameter of 2.6 nm, and thus the feasibility of DNA sequencing using nanopores was proven. Subsequently, the movement of DNA in nanopores was studied using phage Phi29 connector [14] and Aerolysin [15]. Due to some intrinsic problems of biological nanopores, associated with difficulty in preparation, unstable chemical properties and poor repeatability, more and more researchers turned to solid nanopores. The first solid nanopore [16] was synthesized solid on silicon nitride thin films for DNA detection by means of an ion beam in 2001. On the basis of successfully preparing nanopores, other researchers further investigated the movement of DNA using nanopores made of SiO_2_ [17], alumina film [18], graphene nanopore [19], molybdenum disulfide [20], and glass capillary [21,22]. To complement experimental investigations demanding complex preparation processes of solid nanopores and expensive detection equipment, a large number of researchers have applied the continuum model [23,24,25,26,27], molecular dynamics [28,29,30], atomic level Brownian dynamics [31], Brownian kinematics [32] to carry out numerical simulation on particle motion and ion current change in nanopores.

Despite many enlightening reports on particle dynamics through nanopores, theoretical investigations based on numerical simulations deserve further attention. Some key parameters, including the particle length, have not been exploited, and the details of the particle transport mechanism have not been fully elucidated. Herein, a fully coupled continuous model is constructed by the arbitrary Lagrangian−Eulerian (ALE) method. The model is composed of an electric field described by the Poisson equation, a concentration field described by the Nernst−Planck equation, and a flow field described by the Navier−Stokes equation. Based on the model, the electrokinetic mechanism of cylindrical particles is investigated in detail to elucidate the movement of a DNA strand passing through the nanopore. In particular, the effects of the applied electric field intensity and the particle length on the translation velocity in the nanopore and the change in ion current are studied.

## 2. Mathematical Model

**Figure 1:**
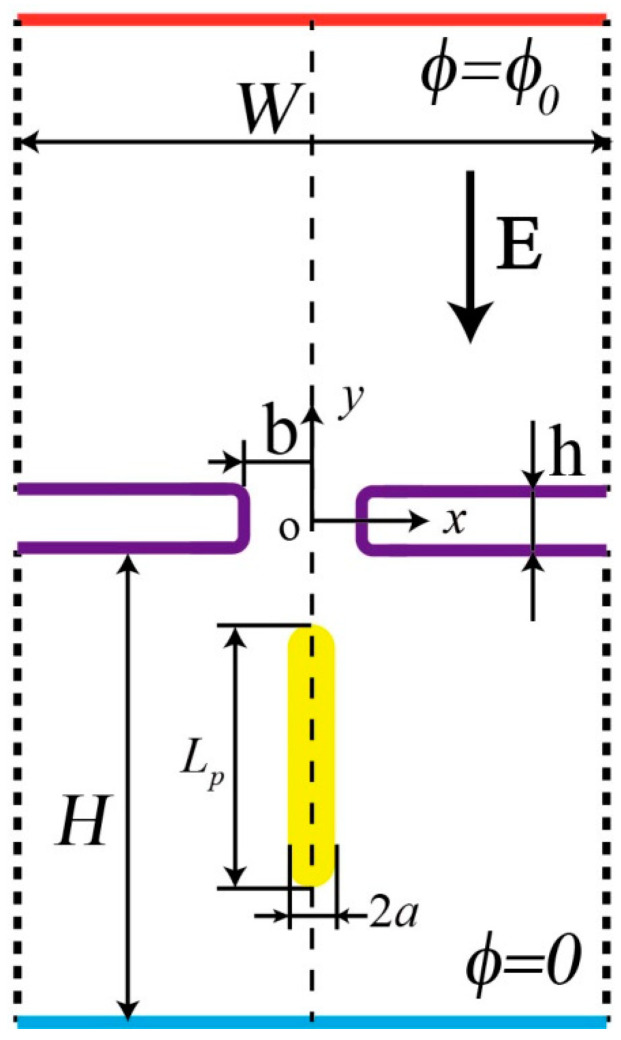
Schematics of a cylindrical particle passing through a nanopore.

We studied a membrane structure with pore diameter *b* and thickness *h*, connecting two nanounits with uniform height *H* and width *W* to form a nanopore channel, as shown in Figure 1. The channel was filled with KCl aqueous solution with concentration *C*_0_, dynamic viscosity μ, the density $\rho_{f}$, and permittivity $\varepsilon_{f}$. A Cartesian coordinate system (x, y) was established at the center of the channel. An electric potential difference was imposed between the upper and lower walls of the nanopore channel, so the ions’ motion in the channel generated current under the action of the DC electric field. A negatively charged cylindrical particle with its length *L*_p_ and the cap radius *a* was put into the nanochannel, the cylindrical particle was defined as a rigid domain, its density was set equal to that of the aqueous solution, the gravity and buoyancy on the particle could be regarded as offsetting each other accordingly, then the particle would move continuously under the action of the electrophoretic force and pass through the nanopore [26]. Since the particle displaced a part of the fluid as it passed through the nanopore, the ions passing through the nanopore decreased, and the ionic current was blocked, inducing a detectable ionic current deviation. The fluctuation magnitude of the current was related to the particle size and the applied electrical field strength.

The electric field distribution in the nanopore is described by the Poisson equation

(1) $$-\varepsilon_{f}\nabla^{2}\phi =F\left(z_{1}c_{1}+z_{2}c_{2}\right)$$

where $\varepsilon_{f}$ is the permittivity of fluid in the nanopore, ϕ is the electric potential, F is the Faraday constant, $z_{i}$ is the valence of the ith ionic species, $c_{i}$ is the concentration of the ith ionic species (i = 1,2), and the right side of the equation represents the bulk charge density of the solution. The potential boundary conditions on the upper and lower walls of the nanopore channel are given as

(2) ϕ(x,−(H+h/2))=0

(3) $$\phi \left(x,\left(H+h/2\right)\right)=\phi_{0}$$

The particle is charged with surface charge density $\sigma_{p}$, which is imposed as a boundary condition on the moving particle surface, where n is the outward unit normal vector on the particle surface. Other rigid boundaries are set to be electrically insulated.

(4) $$-n\cdot \nabla \phi =\;\sigma_{p}$$

The ion distribution in the nanopore is described by the Nernst−Planck equation, expressed as

(5) $$\frac{\partial c_{i}}{\partial t}+\nabla \cdot N_{i}=0,\;i=1\;,\;2$$

(6) $$N_{i}=u_{f}c_{i}-D_{i}\nabla c_{i}-z_{i}\frac{D_{i}}{RT}Fc_{i}\nabla \phi ,\;i=1\;,\;2$$

where $N_{i}$ is the flux of the ith ionic species, $u_{f}$ is the flow velocity in the liquid field, $D_{i}$ is the diffusion coefficient of the ith ionic species, R is the universal gas constant, and T is the absolute temperature of the medium. Each term on the right-hand side of Equation (6) represents the convection, the diffusion, and the electro migration of ionic species, respectively. The ion boundary conditions at the upper and lower walls of the nanopore channel are given as

(7) $$c_{i}\left(x,\pm \left(H+h/2\right)\right)=c_{0},i=1,2$$

The convective flux dominates on the moving particle surface, and thus the normal ionic flux of particle surface is set as

(8) $$n\cdot N_{i}=n\cdot (u_{f}c_{i})$$

There is no ion penetrating the stationary nanopore wall, where the normal flux is set to zero. The upper and lower edges of the two nanounits are set as open boundaries that describe boundaries in contact with a large volume of fluid; fluid can both enter and leave the domain on these boundaries. On the other boundaries, indicated by the dotted lines in Figure 1, no penetration and no shear-stress conditions are prescribed:

(9) $$u_{f}\cdot n=0$$

(10) $$\left[-pΙ+\mu \left(\nabla u_{f}+\nabla u_{f}^{T}\right)\right]\cdot n=0$$

The Navier−Stokes equation is applied to describe the fluid motion in the nanopore. Since the Reynolds number (Re) in the nanopore is extremely small, we can ignore the inertia, and so for an incompressible flow the conservation of momentum and mass are expressed by

(11) $$\rho_{f}\frac{\partial u_{f}}{\partial t}=\nabla \cdot \left[-pΙ+\mu \left(\nabla u_{f}+\nabla u_{f}^{T}\right)\right]+f$$

(12) $$\nabla \cdot u_{f}=0$$

where $\rho_{f}$ is the density of the fluid, $u_{f}$ is the velocity of the flow field, *p* is the fluid pressure, μ is the dynamic viscosity of fluid, and f is the volume force per unit fluid, $f=-F\left(z_{1}c_{1}+z_{2}c_{2}\right)\nabla \phi$, representing the interactive force between the electric field and the net charge in the fluid. The boundary conditions of the upper and lower walls of the nanopore channel are

(13) p(x,±(H+h/2))=0

The particle in the nanopore is forced by both the electric and the flow field, $F_{p}=F_{E}+F_{f}$, where the electric force $F_{E}$ and the hydrodynamic force $F_{f}$ are, respectively, obtained by integrating the Maxwell tensor $\sigma_{E}$ and the hydrodynamic tensor $\sigma_{f}$ on the particle surface $\Gamma_{p}$ as follows:

(14) $$F_{E}=\int \sigma_{E}\cdot nd\Gamma_{p}=\varepsilon_{p}\int \left[EE-\frac{1}{2}\left(E\cdot E\right)I\right]\cdot nd\Gamma_{p}$$

(15) $$F_{f}=\int \sigma_{f}\cdot nd\Gamma_{p}=\int \left[-pI+\mu (\nabla u+\nabla u^{T})\right]\cdot nd\Gamma_{p}$$

where **E** represents the electric field intensity determined by E=−∇ϕ, **I** denotes unit tensor, and $\varepsilon_{p}$ represents the permittivity of the particle. The superscript *T* represents the transpose of the matrix. The particle surface and nanopore wall are set as no slip boundaries, and so the particle surface velocity **u** is identical to the fluid velocity, $u=u_{f}$. The other boundaries are set as symmetrical boundaries.

In addition, the ion current passing through the cross section S of the nanopore is obtained by

(16) $$I=\int_{S}F\left(\sum z_{i}N_{i}\right)\cdot ndS$$

where I is the ion current, and $N_{i}$ is the ionic flux corresponding to the cross section S.

Since the magnitude of ion current *I* passing through the nanopore is very small, the ionic current when the particle is far away from the nanopore is taken as the reference current *I*_0_ (base ionic current), and the dimensionless ion current deviation I* is used to express the difference between them as

(17) $$I^{*}=\left(I-I_{0}\right)/I_{0}$$

I* > 0 means that the ion current passing through the nanopore is greater than the base ionic current, resulting in the ion current enhancement effect, while I* < 0 means that the ion current passing through the nanopore is smaller than the base ionic current, resulting in the ion current blockade effect.

**Figure 2:**
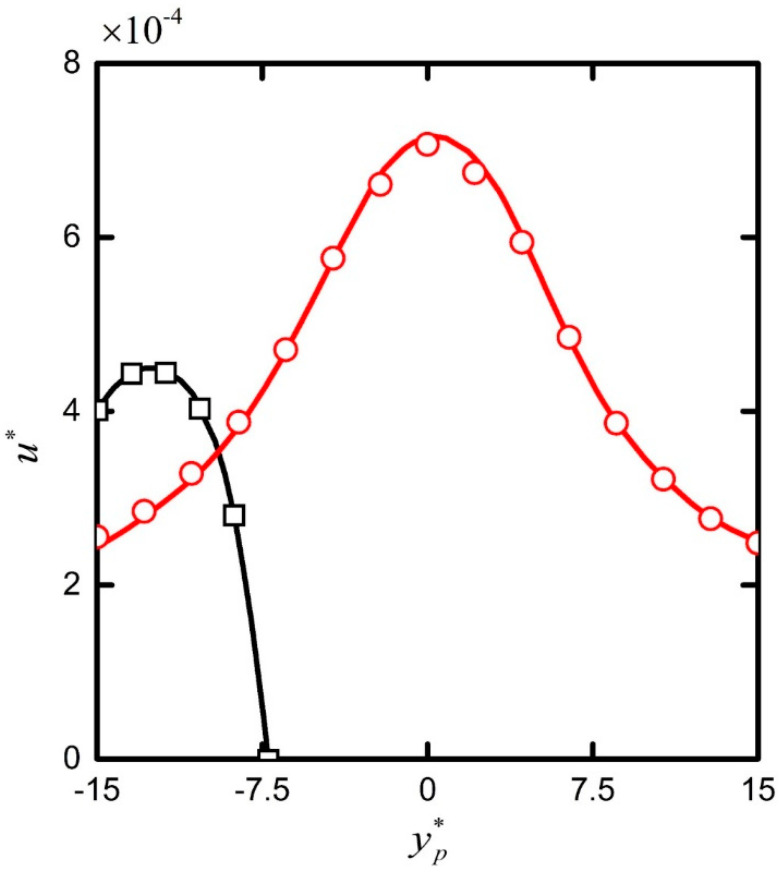
The y-component translational velocity u* of the cylinder particle as a function of the particle’s location y*_p_ under ***E*** = 20 KV/m, the ratio of the particle radius to the Debye length, *ka* = 1.03 (red line and circles), 0.46 (black lines and squares). Symbols and lines represent, respectively, the numerical solution of reference [26] and the numerical results from the present model.

The commercial finite element software COMSOL (version 5.3a, COMSOL Inc., Stockholm, Sweden) was employed to solve the fully coupled electric field, ion transport and particle transport based on the ALE method, which had been proven to be effective in simulating moving boundaries. When the particle started to move (passed the center of the nanopore), the minimum total number of grid and edge elements required were 27,164 (56,900) and 774 (1,357) units, respectively, for convergence, which was also consistent with reference [26]. The model adopted the built-in directive PARDISO solver of COMSOL, with a minimum time step of 8 × 10^−^^7^ [s] and relative tolerance of 0.005 to ensure the convergence of the solution process and the independence of the solution results. In previous reports the integrity of the computational model used was verified by investigating the electrodynamics of deformable or rigid particles inside micro- and nanochannels [33,34,35,36]. Figure 2 additionally shows the high consistency between the numerical simulation used in this study and that in reference [26]. Here we applied this model to further explore the electrokinetic transport mechanism of cylindrical particles in nanopores.

## 3. Results and Discussion

Among many cases studied we presented representative cases with nanounit size *W* = 100 nm and *H* = 200 nm, the membrane structure *h* = 5 nm and *b* = 5 nm, and the radius of the particle cap *a* = 1 nm. The concentration of KCl solution was *C*_0_ = 10 mol/m^3^, while the fluid permittivity, $\varepsilon_{f}=7.08\times 10^{-10}\;F/m$, the permittivity of particle $\varepsilon_{p}$ was set identical to $\varepsilon_{f}$, the solution density $\rho =1\times 10^{3}{\;\mathrm{kg}\;/m}^{3}$, the fluid viscosity $\mu =1\times 10^{-3}\;\mathrm{Pa}\cdot s$, the diffusivity of $K^{+}$, $D_{1}=1.95\times 10^{-9}{\;m}^{2}/s$, and the diffusivity of $Cl^{-}$, $D_{2}=2.03\times 10^{-9}{\;m}^{2}/s$, the solution temperature T=300K, and the particle surface charge density $\sigma_{p}=-0.01C/m^{2}$. Considering that the pitch of the double-helix structure of the common B-DNA molecule is 3.4 nm and 10 base pairs (bp) in one spiral period, we set 1 bp = 0.34 nm. The coordinate variable of particle center a (Xp, Yp) had its initial position (0, −300 bp), below the nanopore 300 bp away from the nanopore center. It should be noted that although the randomly distributed cylindrical particles generally experienced a process of rotating and lateral offset to a position parallel to the electric field prior to entering the nanopore, the orientation of cylindrical particles generally remained unchanged when they approached and entered the nanopore [23,26]. We thus regarded the motion of the cylindrical particles near the nanopore as translational motion, and the velocity u on the particle surface equaled the velocity of the particle as a whole, which simplified the simulation and subsequent analysis. Here we elucidate the translation mechanism of the particle and current fluctuation in the nanopore with focus on the electric field intensity and the length of DNA strand as parameters.

### 3.1. The Effect of Imposed Electric Field

**Figure 3:**
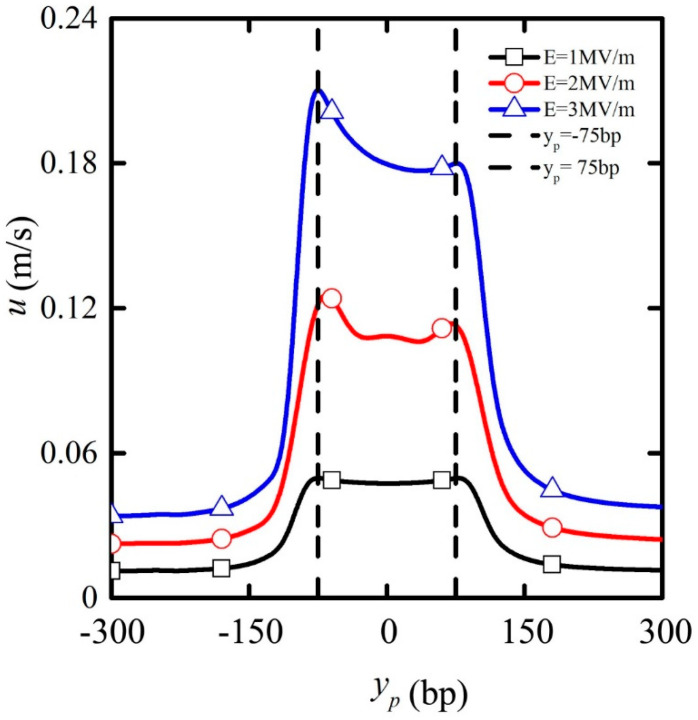
The particle translation velocity depending on particle position under the applied electric field strength of E = 1 MV/m (black line with squares), E = 2 MV/m (red line with circles), and E = 3 MV/m (blue line with triangles) for particle length L_p_ = 200 bp. The vertical dotted lines indicate the locations y_p_ = −75 and y_p_ = 75 bp.

The electric field imposed is a dominant parameter dictating the dynamics of particles by electrophoresis, and so it is necessary to study its effect on particle transport in detail. Since the original position of the cylindrical particle is arranged below the nanopore with no lateral offset with respect to the *y*-axis, the particles always translate along the central axis of the nanopore(see Video S1 in the Supplementary Materials). Figure 3 shows the particle translation velocity depending on the particle position for a particle with length *L*_p_ = 200 bp under the applied electric field strength of E = 1, E = 2 and E = 3 MV/m. Before the particle reached the nanopore, its velocity stayed almost constant. As the particle entered the nanopore, the velocity increased rapidly until it reached a peak value near the position y_p_ = −75 bp, after which it decreased conspicuously until the particle center moved to the nanopore center (y_p_ = 0). As the particle center moved beyond the position y_p_ = 75 bp, its velocity decreased monotonically until it exited the nanopore completely and translated away, as shown in the figure through the position y_p_ = 300 bp. With the increase in the electric field strength, the translation velocity increased consistently during the entire transport process of the particle, and this trend was more pronounced when the particle was located in the mid-section (−75bp < y_p_ < 75bp). The peak values calculated for the applied electric field strength E = 1, E = 2, and E = 3 MV/m were approximately 50, 125, and 210 mm/s, respectively.

**Figure 4:**
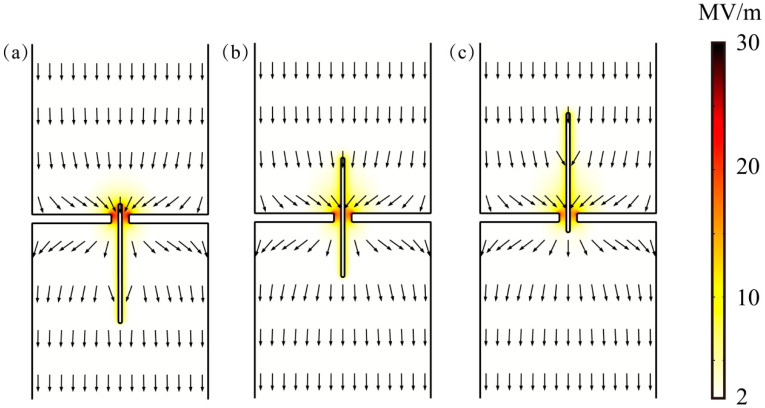
The electric field distribution around the nanopore for particle length L_p_ = 200 bp and E = 2 MV/m when the particle center is located at (**a**) y_p_ = −75, (**b**) y_p_ = 0, (**c**) y_p_ = 75 bp. The darker colors in the diagram represent a stronger electric field, and the arrows indicate the direction of the electric field.

The above velocity profile can be explained by examining the electrophoretic force acting on the particle and electrostatic interaction between the particles and the nanopore. Figure 4 shows the electric field around the nanopore when the particle center was located at y_p_ = −75, y_p_ = 0, y_p_ = 75bp. As shown, strong electric fields were induced around the particle and the nanopore, which was attributed to the formation of the electric double layer (EDL) adjacent to their surfaces. On the one hand, the imposed electric field generated the electrophoretic force compelling the negative charged particles to pass through the nanopore (when the electric polarity of particle or electric field reverses, the particle will move downward rather than through the nanopore, see Video S2 in the Supplementary Materials). On the other hand, the electrostatic interaction was generated when the electric fields around the particles and the nanopore overlapped. When the particle approached the nanopore from below, the attractive electrostatic force between the particles and the nanopore promoted the translation of the particle due to the negative particle surface charge density. Then, when the particle entirely exited the nanopore, the attractive electrostatic force hindered the particle translation, slowing its transport velocity. When the particle was located in the midsection of the nanopore, the distribution of electric field around the cylindrical particle was basically symmetrical, and the electrostatic force on the particles was offset, so that the electrostatic contribution was less pronounced. It is worth mentioning that even if the particles themselves are not charged, if an initial angle between particle axis and the electric field is preset, the particles will rotate rapidly to the direction consistent with the electric field before passing through the nanopore, which can be attributed to the action of the dielectrophoresis force (see Videos S3 and S4 in the Supplementary Materials).

**Figure 5:**
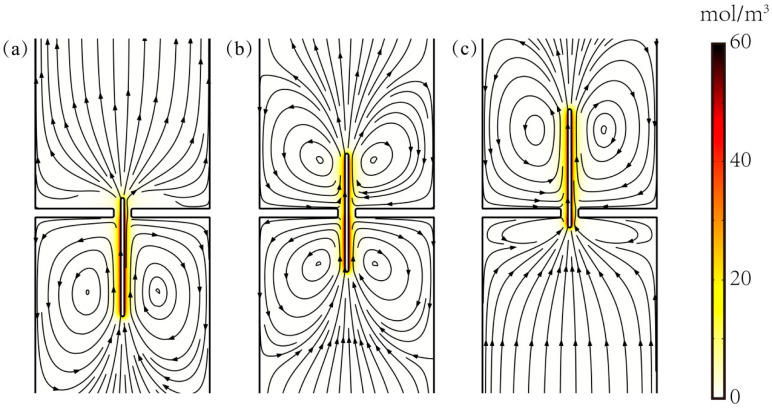
The distribution of the concentration difference c1−c2 between cation and anion and streamline diagram when the particle length is L_p_ = 200 bp, the particle locates at (**a**) y_p_ = −75, (**b**) y_p_ = 0, (**c**) y_p_ = 75 bp. The darker colors in the diagram mean the larger value of c1−c2, and the lines with arrows denote the streamlines of the flow field.

Figure 5 shows the concentration distribution and the streamlines when the particle was located at y_p_ = −75, y_p_ = 0, y_p_ = 75 bp. The color variance in this figure implies the difference between the cation and anion concentration, *c*1–*c*2. From Figure 5, it can be seen that *c*1–*c*2 was almost close to 0 in the nanocavity solution, but there were a lot of cations gathering on the surface of the cylindrical particles due to the predetermined negative charge density on the particle surface. At the same time, the fluid around the particles moved upwards, and when the particles passed through the nanopore, symmetrical vortices were formed on both sides around the particles due to the rapid translation of the particles (see Video S5 in the Supplementary Materials).

**Figure 6:**
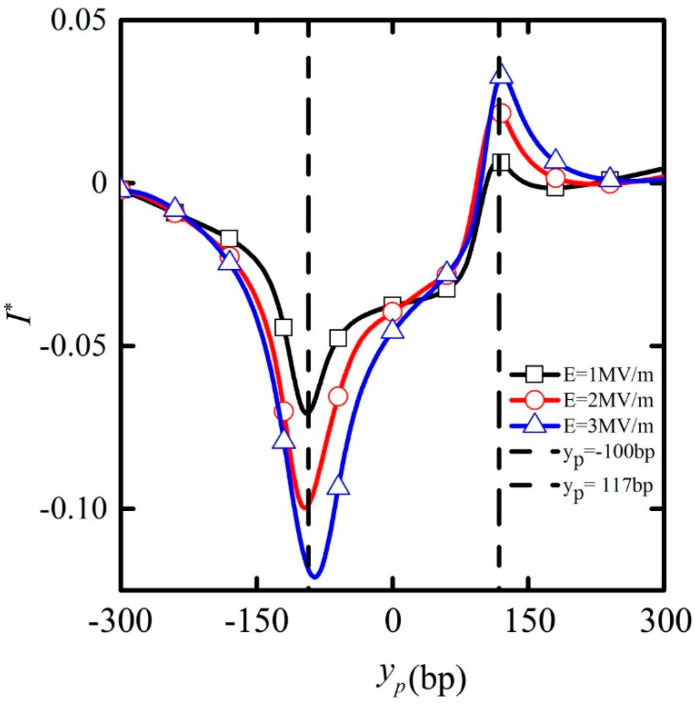
The ion current deviation I^*^ passing through the nanopore depending on the particle position under E = 1 (black line plus frame), E = 2 (red line plus circle), E = 3 MV/m (blue line plus triangle) when the particle length is L_p_ = 200 bp. Dotted lines indicate the particle position y_p_ = −100 and y_p_ = 117 bp, and are not meant to indicate locations for maximum or minimum current.

Figure 6 shows the ionic current deviation I* passing through the nanopore depending on the particle position for E = 1, E = 2, and E = 3 MV/m for a particle length *L* = 200 bp. As the particle started to move, the ionic current deviation I* was almost absent. Considering that dimensionless I* is defined as the difference between the ionic current and the base ionic current in the nanopore, I* = 0 implies that the current in the nanopore equals the base ion current. It then decreased to be negative as the particle approached the nanopore. When the particle moved to y_p_ = −100 bp, the ionic current deviation I* reached its minimum value, which meant that the current in the nanopore was far less than the base ion current. In other words, the current blockade effect occurred. With the continuous translation of the particle in the nanopore, the ionic current deviation I* increased gradually. When the particle moved to y_p_ = 117 bp, I* reached its maximum value, which meant that the current in the nanopore exceeded the base ion current. The current enhancement effect occurred, after which the ionic current decreased gradually. When the particles were far away from the nanopore (y_p_ = 300 bp), the ionic current deviation I* tended to recover zero value, and the current in the nanopore basically recovered to the base ion current. With the increase in the applied electric field, both the ionic current blockade and the enhancement effect were amplified significantly.

When the particle began to enter the nanopore, the ion transport was hindered due to the presence of particles, resulting in the ionic current decrease in the nanopore from the base ionic current, thus the ion current blockade effect. When the particle center arrived at y_p_ = −100 bp, about half of the particle in the nanopore, the degree of the ion blockage reached its maximum. However, with the particle passing through the nanopore, the ionic current in the nanopore was increasing. Because a large number of cations carried on the particle surface flowed out of the nanopore, the ionic current recovered rapidly and even exceeded the base ion current, resulting in the current enhancement effect. In addition, the increase in fluid velocity due to particles passing through the nanopore also increased the ionic current to some extent (see Videos S5 and S6 in the Supplementary Materials). When y_p_ > 117 bp, the entire particle exited the nanopore, the ionic current in the nanopore gradually recovered the base ionic current.

With the increase in the applied electric field, stronger electric fields were induced around the particle and nanopore, which prevented more ions in solution from passing through the nanopores. As a result, the ion blockage effect was promoted. At the same time, more cations were enriched on the surface of the particles under the stronger electric field. As the particles exited the nanopore, the enhancement effect of the ion current was also magnified accordingly.

In summary, both the ionic current blockade and the enhancement effects occurred for the experimental parameters employed in this research. The electric field intensity is an important parameter for adjusting the particle transport velocity and the current fluctuation amplitude. Therefore, if the electric potential or electric field intensity is increased in the experiment, the perforation time of particles could be decreased, thus the transmission efficiency is elevated, which is conducive to the realization of high-throughput sequencing of particles. The increase in the electric field intensity could also significantly increase the fluctuation range of the ionic current, which would reduce the requirements for the sensitivity of current detection instruments, accommodating improvements in the detectability of ionic current.

### 3.2. The Effect of Cylindrical Particle Length

**Figure 7:**
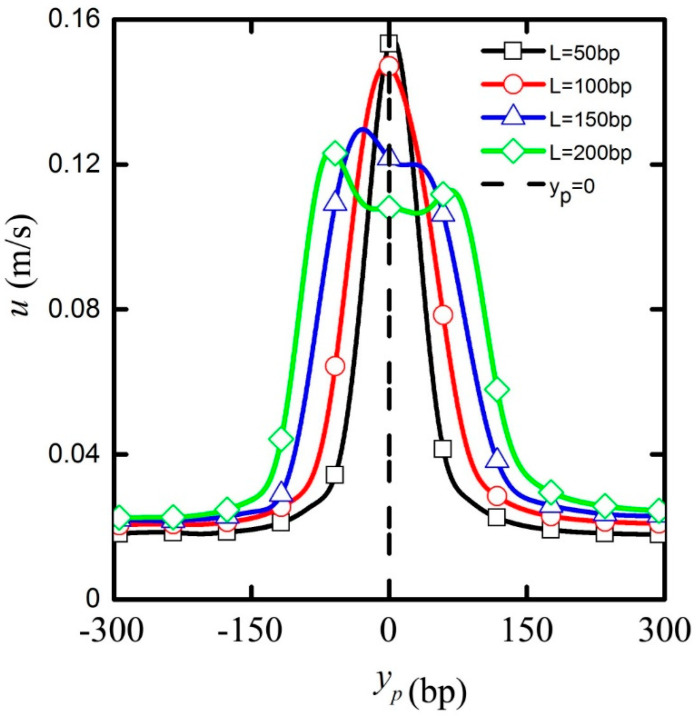
The particle translation velocity vs. particle position for E = 2 MV/m and particle length *L*_p_ = 50 (black line plus frame), *L*_p_ = 100 (red line plus circle), *L*_p_ = 150 (blue line plus triangle), and *L*_p_ = 200 bp (green line plus diamond). The vertical dotted line indicates the center of the nanopore.

Since the electrostatic interaction between the nanopore and the particle is significant only when the particle is located inside the nanopore, the particle length *L*_p_ is another parameter of importance to study in order to further explain the detailed mechanism of the particle dynamics. Figure 7 shows the relationship between transport velocity and the particle position for particle length *L*_p_ = 50, *L*_p_ = 100, *L*_p_ = 150, *L*_p_ = 200 bp under the applied electric field intensity of E = 2 MV/m. It can be seen that when the particle was relatively short (*L*_p_ = 50 and *L*_p_ = 100 bp), the region for constant transport velocity before the nanopore was further extended toward the nanopore. The rapid velocity increase at the entrance of the nanopore culminated in a peak value at a location closer to the midsection near y_p_ = 0 as opposed to the premature peak location shown in Figure 3. The peak value increased with the decrease in the particle length. The velocity decay beyond the midsection was more rapid, and so the recovery of the initial velocity seemed to occur nearer to the nanopore. With the increase in the particle length (*L*_p_ = 150 bp and *L*_p_ = 200 bp), the maximum transport velocity of particles was reduced, and the particle position corresponding to the maximum transport velocity shifted toward the nanopore entrance. The monotonic acceleration and deceleration shown for shorter particles were replaced by more complicated fluctuations in the particle velocity inside the nanopore.

The above velocity change was also attributed to the electrostatic interaction between the nanopore and the particle for different lengths. When the particle is shorter, a larger part of it is dominated by the electric field around the nanopore. When the particle length is longer, only a small part of it is affected by the electric field around the nanopore. As stated before, when the particle is located in the midsection of the nanopore, the electric field distribution is symmetrical with respect to the cylindrical particle, and the electrostatic force acting on the particles is basically offset, so that the particle’s velocity remains basically unchanged. With the increase in the particle length, its center position as the particle enters the nanopore is pushed downward leading to that fact the particle’s position corresponding to the maximum transport velocity is also pushed downward and the acceleration distance and the maximum transport velocity of particles is reduced.

**Figure 8:**
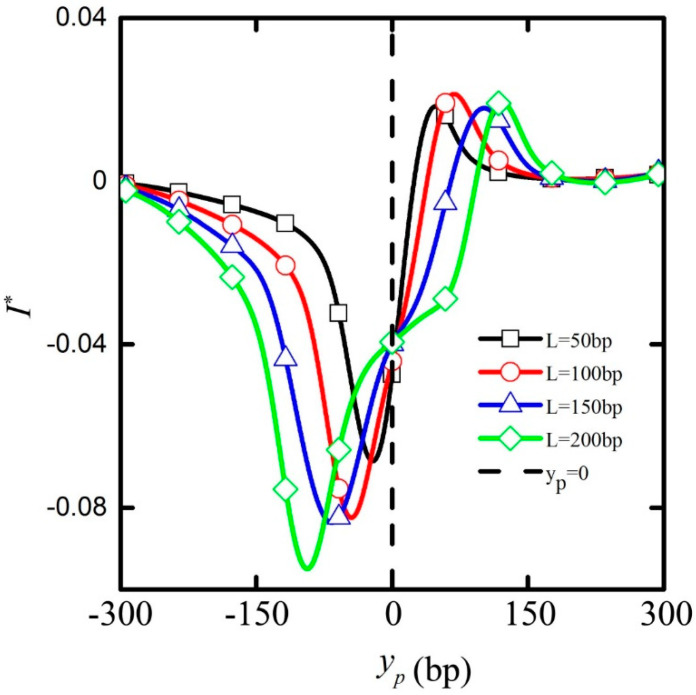
The ion current deviation I^*^ passing through the nanopore depending on the particle position when the applied electric field strength E = 2 MV/m, the particle length is *L*_p_ = 50 (black line plus frame), *L*_p_ = 100 (red line plus circle), *L*_p_ = 150 (blue line plus triangle), and *L*_p_ = 200 bp (green line plus diamond). The dotted line indicates that the particles reach the center of the nanopore, y_p_ = 0.

Figure 8 shows the ionic current deviation I* passing through the nanopore depending on the particle position for electric field E = 2 MV/m and particle length *L*_p_ = 50, *L*_p_ = 100, *L*_p_ = 150, and *L*_p_ = 200 bp. It can be seen that both the ionic current blockage and enhancement effects occurred when these cylindrical particles passed through the nanopore, which was consistent with previous research [24,26]. With the increase in the particle length, the phenomenon of the ionic current blockage was amplified, whereas the ionic current enhancement effect had little change. At the same time, the particle position corresponding to the ion current blockage was pushed downward, while the particle position corresponding to the ion current enhancement was pushed upward. In addition, when particles with different lengths reached the center of the nanopore y_p_ = 0, almost identical ion currents passing through the nanopore were obtained.

With the increase in the particle length, more space was occupied by the particle in the nanopore, and the degree of blockade of ion transport was aggravated. The effect of the ionic blockade thus was magnified. Moreover, the particle center position y_p_ shifted downward, as the upper end of the particle entered the nanopore, which led to the fact that the y_p_ corresponding to ion current blockage effect was pushed downward accordingly. Similarly, the y_p_ shifted upward, as the lower end of the particle exited the nanopore, and the y_p_ corresponding to the ion current enhancement effect was pushed upward. However, when the particle was located in the center of the nanopore, the degree of blockade of ion transport had little difference for particles with different lengths, which made the ion currents passing through the nanopore remain almost the same.

In summary, the increase in the particle length gives rise to a decrease in the peak value of particle translation velocity. The positions of the ion current peak values corresponding to particles with different lengths are also different, which is helpful in distinguishing particles with different lengths by detecting the ionic current peak values and their corresponding residence time.

## 4. Conclusions

In this study, fully coupled Poisson−Nernst−Planck and Navier−Stokes equations are solved by an arbitrary Lagrangian−Eulerian (ALE) method to investigate the transport mechanism of cylindrical particles in a nanopore. In particular, the influence of an applied electric field and the particle length on the transport of cylindrical particles in nanopores is studied. The results show that the acceleration and deceleration of the cylindrical particles inside the entrance and exit regions of the nanopore become less pronounced with the increase in the particle length. The translation velocity for long particles is maintained relatively uniform inside the nanopore. The ionic current blockade effect occurs as particles enter the nanopore, and the ionic current enhancement effect occurs as they exit the nanopore for the parameter ranges studied. As an important parameter to control the particle transport velocity and current fluctuation amplitude, the electric field intensity can be used to adjust the particle transmission efficiency and ion current detectability. The increase in particle length reduces the peak value of particle velocity and changes the particle position corresponding to the peak value of the ion current, which contributes to distinguishing particles with different lengths. The results of this study deepen the understanding of the transport mechanism of long cylindrical particles in nanopores, with potential applications to bio-macromolecule detection technology.

## Supplementary Materials

Supplementary materials can be found at https://www.mdpi.com/2072-666X/11/8/722/s1. Video S1: The electric field fluctuation as the particle passes through nanopore; Video S2: The particle moves downward at the case of the reversed electric field; Video S3: The particle rotates clockwise to the direction of the electric field (It should be noted that the particle itself is not charged, the round cap of the particle is removed to facilitate the drawing); Video S4: The particle rotates counter-clockwise to the direction of the electric field. (It should be noted that the particle itself is not charged, the round cap of the particle is removed to facilitate the drawing); Video S5: The fluid velocity field fluctuation as the particle passes through nanopore; Video S6: The fluid pressure field fluctuation as the particle passes through nanopore.
