# Supplementary material for: The Influence of Electric Field Intensity and Particle Length on the Electrokinetic Transport of Cylindrical Particles Passing through Nanopore
Source: Micromachines (Basel). 2020 Jul 25;11(8):722. doi: 10.3390/mi11080722 (PMC7463976; doi:10.3390/mi11080722)
Supplement: Supplementary file 1 [file micromachines-11-00722-s001.zip › micromachines-831976 supplementary/Supplymentary file-caption of Video.docx]

Caption of Video 1-6

Video 1: The Electric field fluctuation during particles passing through nanopores.

Video 2: Particles move downward at the case of the reversed Electric field.

Video 3: The particle rotation clockwise to the direction of the electric field(It should be noted that the particles themselves are not charged,the round caps of the particles are removed to facilitate the drawing ).

Video 4: The particle rotation counter-clockwise to the direction of the electric field. (It should be noted that the particles themselves are not charged, the round caps of the particles are removed to facilitate the drawing)

Video 5: The fluid velocity field fluctuation during particles passing through nanopores.

Video 6: The fluid pressure field fluctuation during particles passing through nanopores.
