# Supplementary figures and images for: The Influence of Electric Field Intensity and Particle Length on the Electrokinetic Transport of Cylindrical Particles Passing through Nanopore
Source: Micromachines (Basel). 2020 Jul 25;11(8):722. doi: 10.3390/mi11080722 (PMC7463976; doi:10.3390/mi11080722)

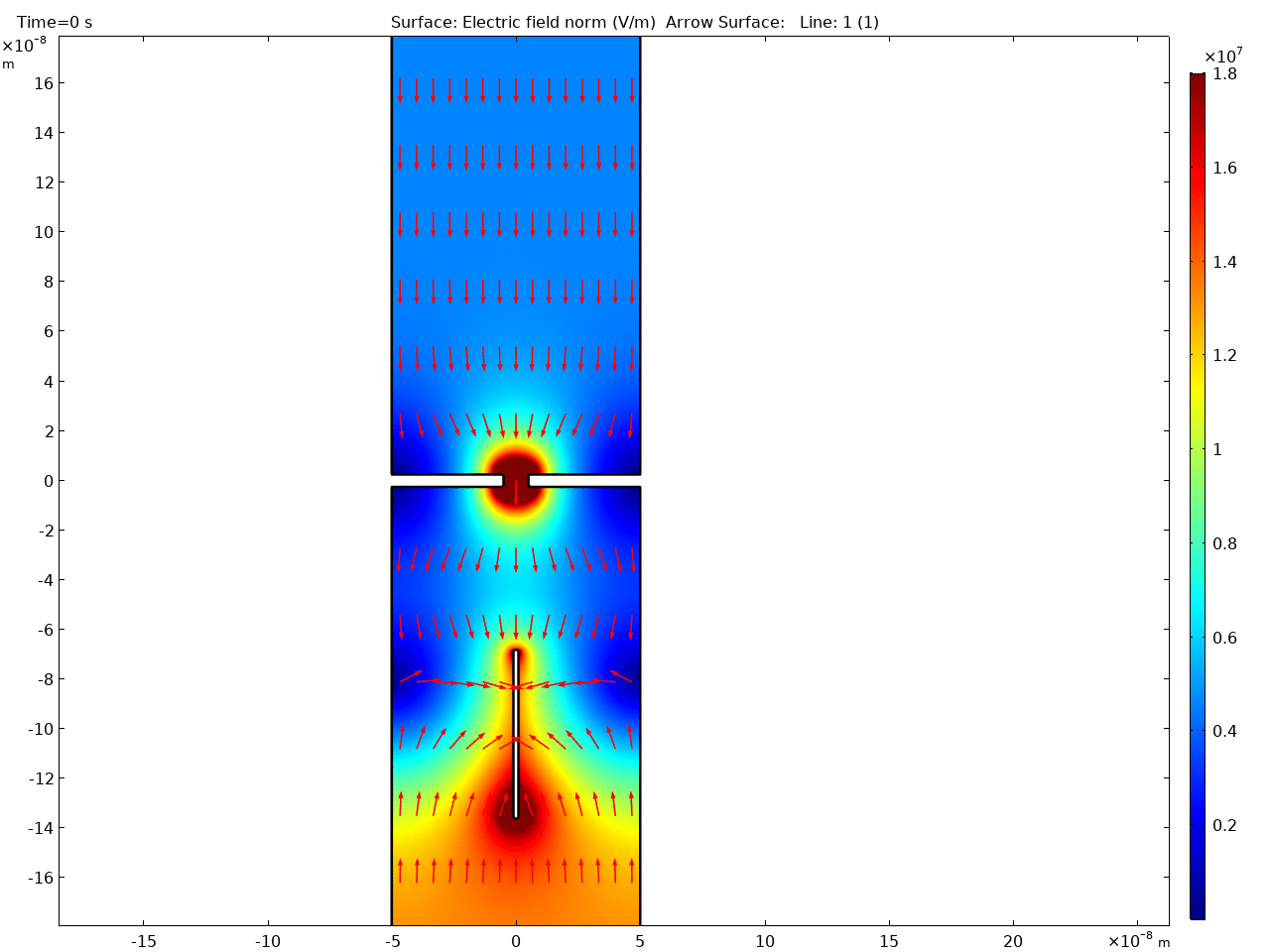

Supplement: Supplementary file 1 [file micromachines-11-00722-s001.zip › micromachines-831976 supplementary/Video S1.gif]

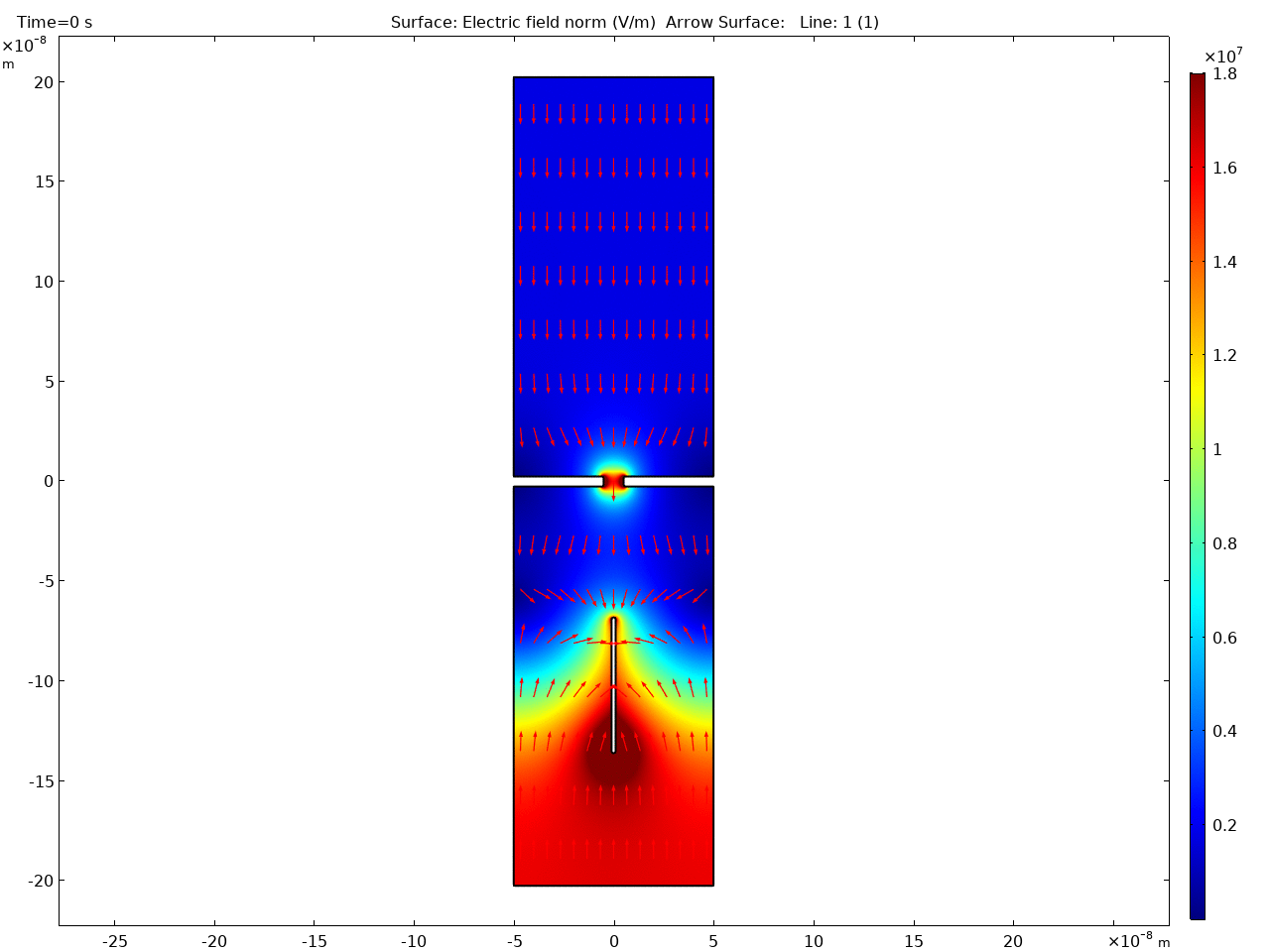

Supplement: Supplementary file 1 [file micromachines-11-00722-s001.zip › micromachines-831976 supplementary/Video S2.gif]

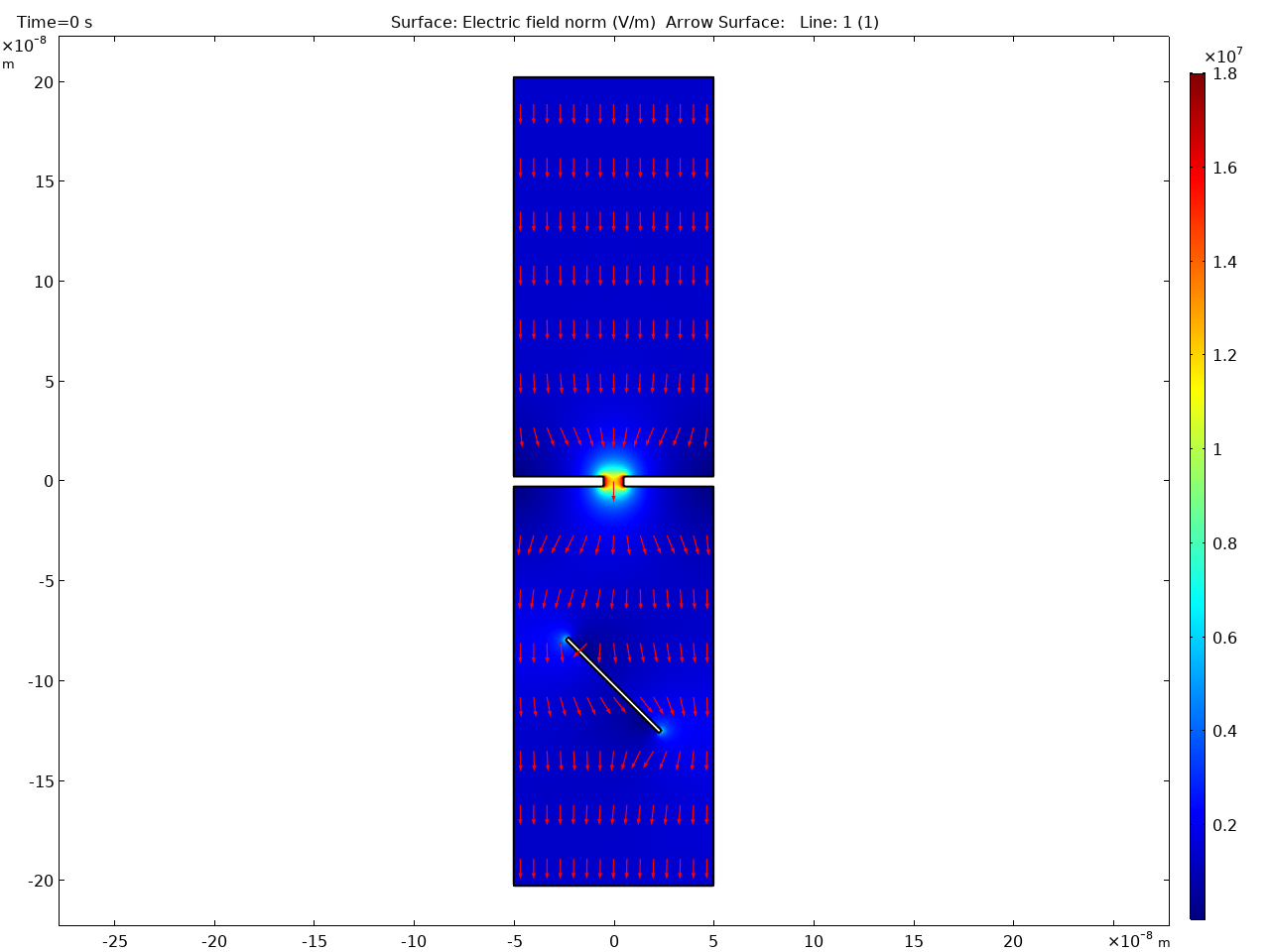

Supplement: Supplementary file 1 [file micromachines-11-00722-s001.zip › micromachines-831976 supplementary/Video S3.gif]

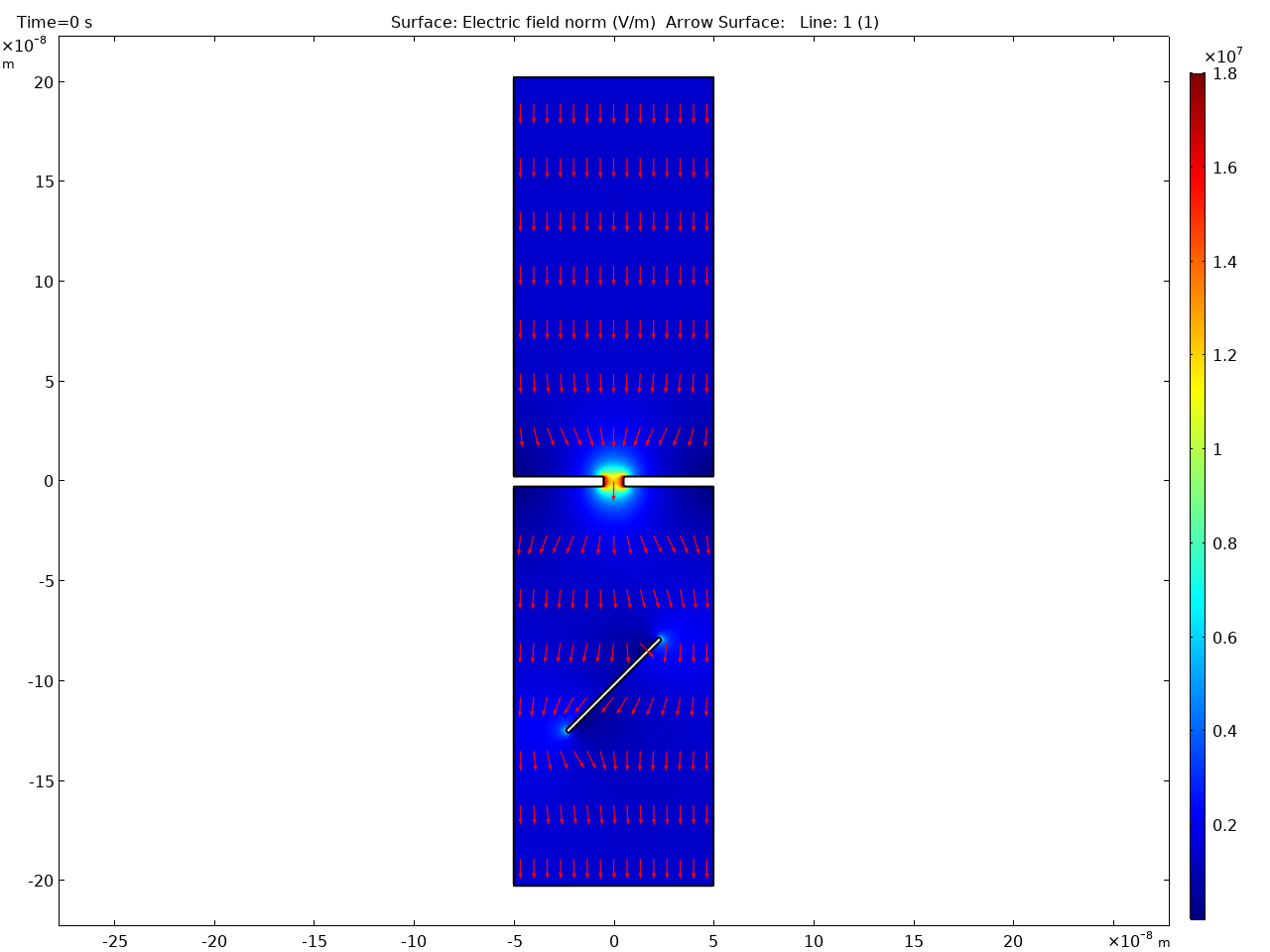

Supplement: Supplementary file 1 [file micromachines-11-00722-s001.zip › micromachines-831976 supplementary/Video S4.gif]

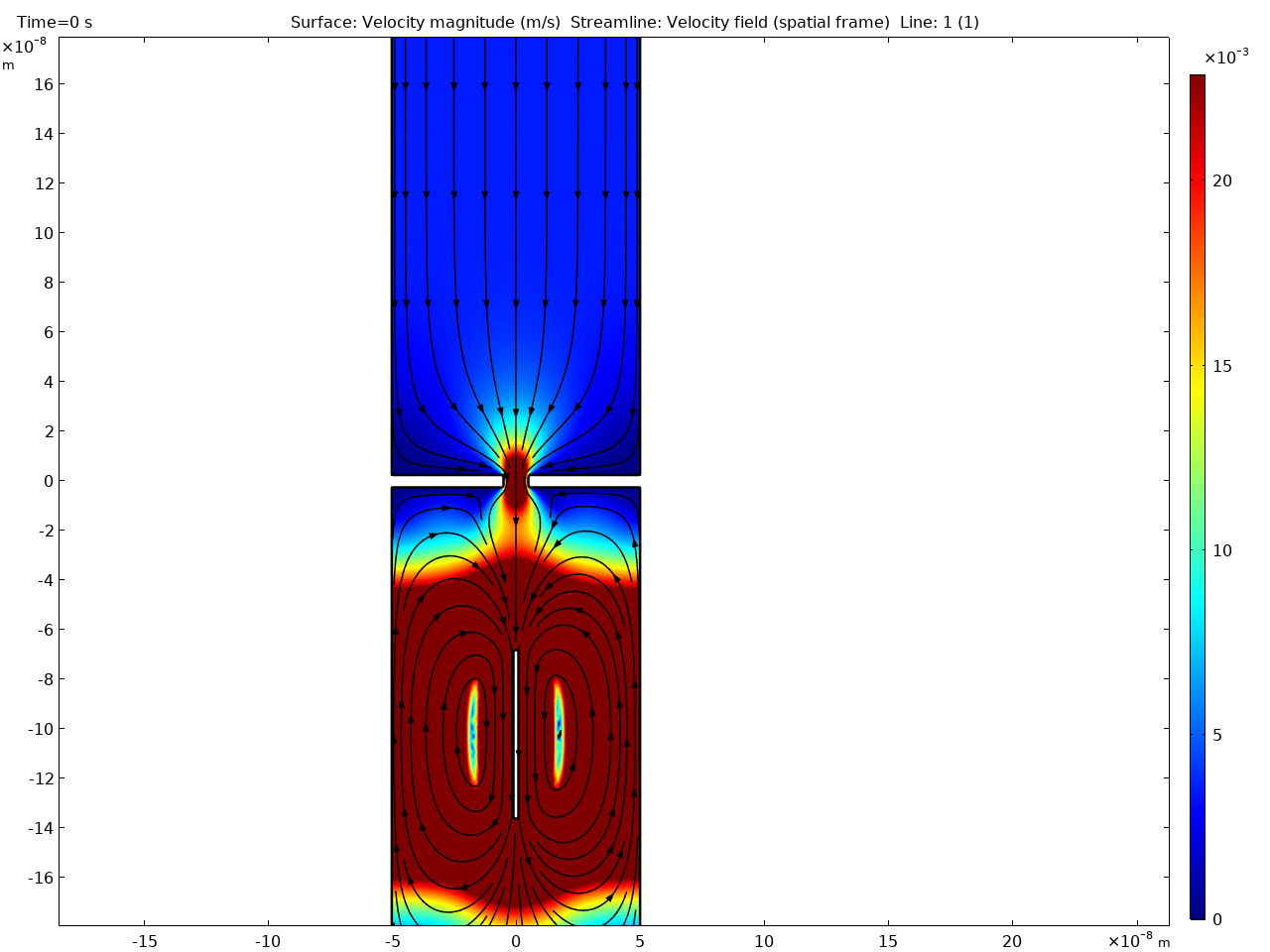

Supplement: Supplementary file 1 [file micromachines-11-00722-s001.zip › micromachines-831976 supplementary/Video S5.gif]

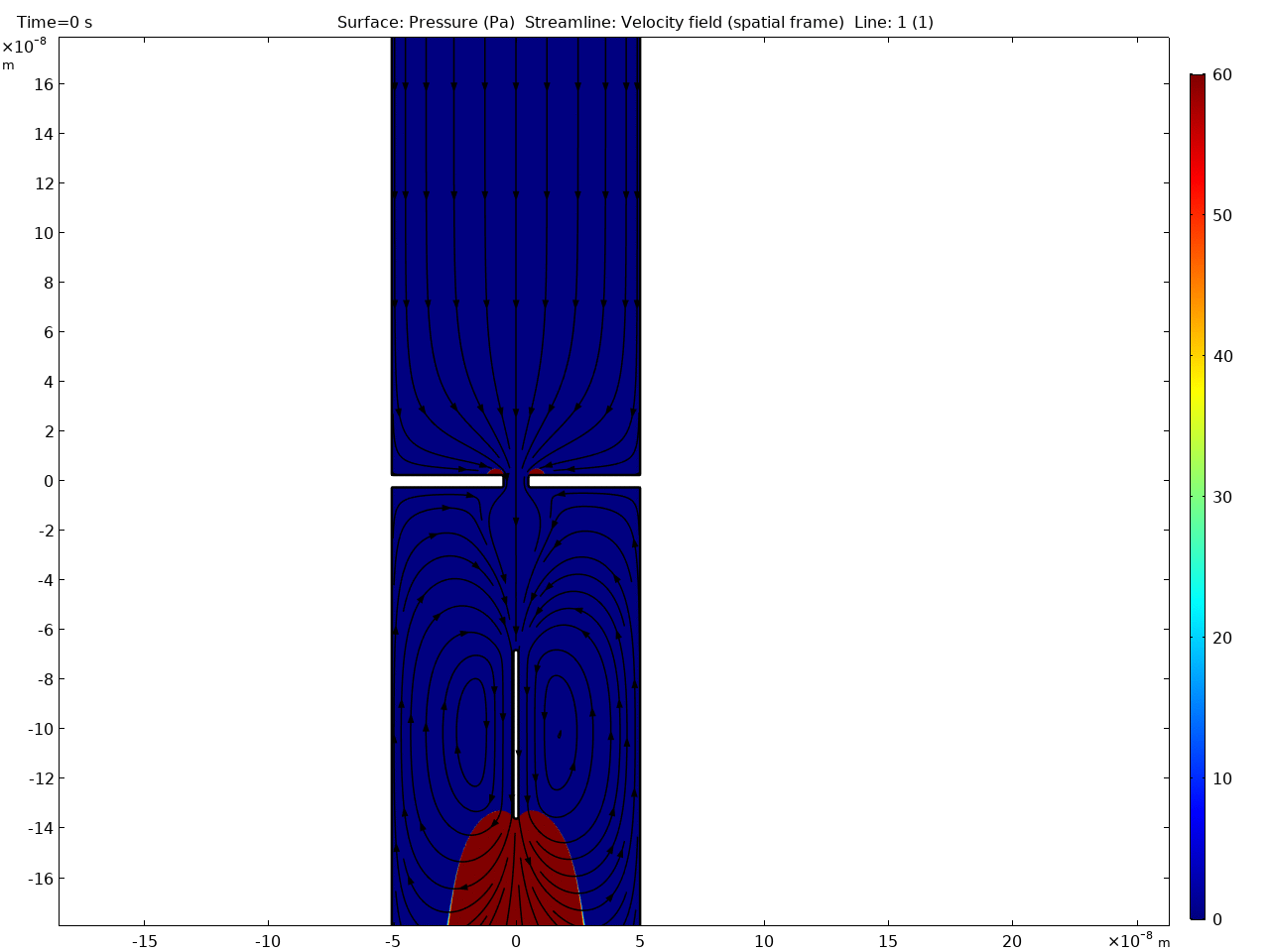

Supplement: Supplementary file 1 [file micromachines-11-00722-s001.zip › micromachines-831976 supplementary/Video S6.gif]
